# Potential contribution of HIV during first-line tuberculosis treatment to subsequent rifampicin-monoresistant tuberculosis and acquired tuberculosis drug resistance in South Africa: a retrospective molecular epidemiology study

**DOI:** 10.1016/S2666-5247(21)00144-0

**Published:** 2021-11

**Authors:** Helen Cox, Zubeida Salaam-Dreyer, Galo A Goig, Mark P Nicol, Fabrizio Menardo, Anzaan Dippenaar, Erika Mohr-Holland, Johnny Daniels, Patrick G T Cudahy, Sonia Borrell, Miriam Reinhard, Anna Doetsch, Christian Beisel, Anja Reuter, Jennifer Furin, Sebastien Gagneux, Robin M Warren

**Affiliations:** a Division of Medical Microbiology, Department of Pathology, University of Cape Town, Cape Town, South Africa; b Institute of Infectious Disease and Molecular Medicine, Wellcome Centre for Infectious Disease Research, University of Cape Town, Cape Town, South Africa; c Swiss Tropical and Public Health Institute, Basel, Switzerland; d University of Basel, Basel, Switzerland; e Division of Infection and Immunity, School of Biomedical Sciences, University of Western Australia, Perth, WA, Australia; f Tuberculosis Omics Research Consortium, Family Medicine and Population Health, Institute of Global Health, Faculty of Medicine and Health Sciences, University of Antwerp, Antwerp, Belgium; g Médecins Sans Frontières, Khayelitsha, Cape Town, South Africa; h Section of Infectious Diseases, Department of Internal Medicine, Yale School of Medicine, New Haven, CT, USA; i Department of Global Health and Social Medicine, Harvard Medical School, Boston, MA, USA; j DST-NRF Centre of Excellence for Biomedical Tuberculosis Research/SAMRC Centre for Tuberculosis Research, Division of Molecular Biology and Human Genetics, Faculty of Medicine and Health Sciences, Stellenbosch University, Stellenbosch, South Africa

## Abstract

**Background:**

South Africa has a high burden of rifampicin-resistant tuberculosis (including multidrug-resistant [MDR] tuberculosis), with increasing rifampicin-monoresistant (RMR) tuberculosis over time. Resistance acquisition during first-line tuberculosis treatment could be a key contributor to this burden, and HIV might increase the risk of acquiring rifampicin resistance. We assessed whether HIV during previous treatment was associated with RMR tuberculosis and resistance acquisition among a retrospective cohort of patients with MDR or rifampicin-resistant tuberculosis.

**Methods:**

In this retrospective cohort study, we included all patients routinely diagnosed with MDR or rifampicin-resistant tuberculosis in Khayelitsha, Cape Town, South Africa, between Jan 1, 2008, and Dec 31, 2017. Patient-level data were obtained from a prospective database, complemented by data on previous tuberculosis treatment and HIV from a provincial health data exchange. Stored MDR or rifampicin-resistant tuberculosis isolates from patients underwent whole-genome sequencing (WGS). WGS data were used to infer resistance acquisition versus transmission, by identifying genomically unique isolates (single nucleotide polymorphism threshold of five). Logistic regression analyses were used to assess factors associated with RMR tuberculosis and genomic uniqueness.

**Findings:**

The cohort included 2041 patients diagnosed with MDR or rifampicin-resistant tuberculosis between Jan 1, 2008, and Dec 31, 2017; of those, 463 (22·7%) with RMR tuberculosis and 1354 (66·3%) with previous tuberculosis treatment. In previously treated patients, HIV positivity during previous tuberculosis treatment versus HIV negativity (adjusted odds ratio [OR] 2·07, 95% CI 1·35–3·18), and three or more previous tuberculosis treatment episodes versus one (1·96, 1·21–3·17) were associated with RMR tuberculosis. WGS data showing MDR or rifampicin-resistant tuberculosis were available for 1169 patients; 360 (30·8%) isolates were identified as unique. In previously treated patients, RMR tuberculosis versus MDR tuberculosis (adjusted OR 4·96, 3·40–7·23), HIV positivity during previous tuberculosis treatment (1·71, 1·03–2·84), and diagnosis in 2013–17 (1·42, 1·02–1·99) versus 2008–12, were associated with uniqueness. In previously treated patients with RMR tuberculosis, HIV positivity during previous treatment (adjusted OR 5·13, 1·61–16·32) was associated with uniqueness as was female sex (2·50 [1·18–5·26]).

**Interpretation:**

These data suggest that HIV contributes to rifampicin-resistance acquisition during first-line tuberculosis treatment and that this might be driving increasing RMR tuberculosis over time. Large-scale prospective cohort studies are required to further quantify this risk.

**Funding:**

Swiss National Science Foundation, South African National Research Foundation, and Wellcome Trust.

## Introduction

Globally, an estimated half a million individuals develop rifampicin-resistant tuberculosis annually; among these, 82% have multidrug-resistant (MDR) tuberculosis with resistance to both rifampicin and isoniazid. The remaining 18% have rifampicin-monoresistant (RMR) tuberculosis, defined as rifampicin resistance and isoniazid susceptibility.^1^ In South Africa, more than 13 000 individuals were diagnosed with MDR or rifampicin-resistant tuberculosis in 2019, placing South Africa in the top ten high-burden countries.^1^ Data from two national surveys done in 2001–02 and 2012–14 suggest that although MDR tuberculosis has remained at approximately 3% of all tuberculosis cases across the decade, the proportion of RMR tuberculosis has increased from 0·5% to 1·8%, now comprising 38% of all rifampicin-resistant tuberculosis.^2^

Individuals might develop MDR or rifampicin-resistant tuberculosis through either direct transmission of an already drug-resistant *Mycobacterium tuberculosis* strain, or the acquisition of resistance during first-line tuberculosis treatment. Modelling data describing the relative contributions of direct transmission and resistance acquisition to the MDR or rifampicin-resistant tuberculosis burden are conflicting. Some suggest that more than 90% of all MDR tuberculosis cases in high-burden settings are due to transmission,^3^ although other models suggest that a significant proportion of the MDR or rifampicin-resistant tuberculosis burden is caused by acquired resistance.^3^, ^4^ In South Africa, up to 30% of resistance might be acquired, with this proportion expected to remain at approximately 20% until 2040.^4^

**Research in context**

**Evidence before this study**

Although there is evidence of association between HIV infection and drug-resistant tuberculosis, it is not clear whether this association is driven by increased transmission of drug-resistant tuberculosis to HIV-positive individuals or increased risk of tuberculosis resistance acquisition during tuberculosis treatment in individuals who are HIV-positive compared with HIV-negative individuals. We searched PubMed, EBSCOHost, Scopus, and Web of Science, on Jan 6, 2020, for reports describing associations between rifampicin-resistant tuberculosis (including rifampicin-monoresistant [RMR] tuberculosis and multidrug-resistant [MDR] tuberculosis), HIV, and resistance acquisition or transmission. Only full text articles in English were reviewed. Several studies suggest that HIV is associated with rifampicin-resistant tuberculosis in individuals who have never been treated for tuberculosis before, suggesting direct transmission. However, data also suggest that there is a link between HIV and tuberculosis drug resistance in individuals previously treated for tuberculosis. These data are supported by small case series showing increased acquisition of rifampicin resistance, resulting in RMR tuberculosis, in HIV-positive individuals, particularly those who are severely immunocompromised. Although epidemiological studies from low tuberculosis burden settings suggest that individuals with RMR tuberculosis were more likely to be HIV-positive at diagnosis and to have been previously treated for tuberculosis, no large-scale studies in high tuberculosis and HIV burden settings assessing the risk of acquired resistance during tuberculosis treatment by HIV status were identified.

**Added value of this study**

This is the first report describing an increased risk of both RMR tuberculosis and acquisition of tuberculosis drug resistance in individuals who were HIV-positive during previous tuberculosis treatment compared with those who were HIV-negative. Given the low risk of resistance acquisition on an individual basis, retrospective analysis of a large dataset containing all individuals diagnosed with rifampicin-resistant tuberculosis in a subdistrict of Cape Town, South Africa, over a decade linked with whole-genome sequencing data from matching tuberculosis isolates, allowed estimation of the contribution of HIV infection to resistance acquisition. Additionally, the use of a health data exchange, drawing on public sector disease register, health-care facility, and laboratory data, in this high tuberculosis and HIV burden setting, enabled the inclusion of detailed and complete data on HIV status during previous tuberculosis treatment, adding a novel dimension to the analysis. We have shown that in more than 1300 individuals, those who were HIV-positive during previous tuberculosis treatment were twice as likely to have RMR tuberculosis compared with MDR tuberculosis. Among 164 individuals previously treated for tuberculosis with RMR tuberculosis, those who were HIV-positive during previous tuberculosis treatment were five times more likely to have genomically unique tuberculosis isolates compared with those who were HIV-negative, suggesting resistance acquisition rather than direct transmission.

**Implications of all the available evidence**

These data suggest that treatment of tuberculosis in people living with HIV results in an increased risk of rifampicin resistance acquisition. Although this risk is likely to be small at an individual level, given the large burden of HIV and tuberculosis co-infection in countries like South Africa, any increased risk could continue to fuel the drug-resistant tuberculosis epidemic. Further work on increased rifampicin dosing and optimisation of first-line tuberculosis treatment for HIV-positive individuals is required.

In South Africa, an estimated 7·7 million people are living with HIV and 58% of all individuals with tuberculosis are HIV-positive.^1^ Although HIV has undoubtedly driven the broader tuberculosis epidemic, systematic reviews also suggest an independent association between HIV and MDR or rifampicin-resistant tuberculosis.^5^, ^6^ This association could be due to either increased resistance acquisition or increased transmission of resistant strains among people living with HIV. This group experiences a disproportional influence of nosocomial transmission, particularly in high HIV prevalence settings where exposure to MDR or rifampicin-resistant tuberculosis and increased vulnerability to tuberculosis infection due to HIV are likely to co-exist in health-care facilities.

HIV can also be specifically associated with increased resistance acquisition,^7^ potentially due to HIV-related pharmacokinetic effects, such as malabsorption of tuberculosis drugs, an effect particularly pronounced for rifampicin.^8^ A strong association between HIV and RMR tuberculosis in some settings supports this hypothesis.^9^, ^10^ In people living with HIV, acquired rifampicin resistance has been associated with advanced HIV-related immunosuppression and high bacterial burden due to disseminated tuberculosis disease.^11^

Data from our South African setting shows significant differences between RMR tuberculosis and MDR tuberculosis in terms of rifampicin-resistance conferring mutations and resistance to other tuberculosis drugs (unpublished data). We hypothesised that HIV infection during first-line tuberculosis treatment is a potential driver of either RMR tuberculosis, or resistance acquisition, or both. To address this hypothesis, we did a retrospective cohort study in patients diagnosed with MDR or rifampicin-resistant tuberculosis in Khayelitsha, Cape Town, South Africa.

## Methods

### Study design

This was a retrospective cohort study of individuals routinely diagnosed with MDR or rifampicin-resistant tuberculosis in Khayelitsha, Cape Town, between Jan 1, 2008, and Dec 31, 2017. Reporting of this study follows the STROME-ID guidance. Data were derived from a prospectively maintained database as previously described,^12^ with additional data on previous tuberculosis treatment and HIV infection during previous tuberculosis treatment accessed from the Western Cape Provincial Health Data Centre (PHDC).^13^ To assess resistance acquisition, available *M tuberculosis* isolates stored in a prospectively maintained biobank in the Western Cape, South Africa, from patients in the main cohort underwent whole-genome sequencing (WGS). WGS data were used to define genomically unique MDR or rifampicin-resistant tuberculosis isolates, based on the assumption that unique isolates are more likely to represent acquired drug resistance.^14^ Storage of MDR or rifampicin-resistant tuberculosis isolates in the biobank was approved by the Stellenbosch University ethics committee (N09/11/296), and linkage of WGS data to patient clinical data was approved by the University of Cape Town ethics committee (HREC 416/2014). Permission to access PHDC data was granted by the Western Cape Department of Health.

### Study setting and cohort selection

Khayelitsha is a predominantly poor, peri-urban subdistrict in metropolitan Cape Town. Among the population of approximately 400 000 people, antenatal HIV prevalence is 34% and tuberculosis case notification is 1600 per 100 000 people per year, with tuberculosis and HIV co-infection of more than 70%. Since 2010, the number of patients diagnosed with MDR or rifampicin-resistant tuberculosis per year has remained consistent at approximately 180–200, resulting in an estimated MDR or rifampicin-resistant tuberculosis incidence of 80 cases per 100 000 per year. Before 2011, only patients with tuberculosis at high risk for MDR or rifampicin-resistant disease were tested for tuberculosis drug resistance; subsequently all patients with presumed tuberculosis are tested with Xpert MTB/RIF (Cepheid, Sunnyvale, CA, USA), with further drug susceptibility testing as per national guidance. Data were extracted from the database to form the study cohort, which included all patients with microbiologically confirmed MDR or rifampicin-resistant tuberculosis with a first rifampicin-resistant tuberculosis diagnosis date between Jan 1, 2008, and Dec 31, 2017. Patients without routine drug susceptibility testing for isoniazid were excluded. A subset of this cohort, based on the availability of WGS data from stored MDR or rifampicin-resistant tuberculosis isolates, was used to assess resistance acquisition.

### Determination of HIV positivity during previous tuberculosis treatment

Previous first-line tuberculosis treatment and details of previous tuberculosis treatment episodes (dates, outcomes) were recorded on the MDR or rifampicin-resistant tuberculosis database in Khayelitsha.^12^ These data were supplemented by data from the PHDC, which draws together data from patient administration systems in all fixed public sector health facilities, using unique patient health identifiers.^13^ Based on both routinely recorded and PHDC data, we determined whether patients had received any previous first-line tuberculosis treatment, and for those who were HIV-positive at MDR or rifampicin-resistant tuberculosis diagnosis, we determined the date that the patient was first known to be HIV-positive. This included data from enrolment into HIV care, CD4 cell count testing, and initiation of antiretroviral therapy (ART). Any patient who had at least one previous tuberculosis treatment episode, defined as registration in the tuberculosis treatment register or supply of first-line tuberculosis drugs, after they were known to be HIV-positive, was classified as HIV-positive during previous tuberculosis treatment.

### WGS and inference of acquired drug resistance

Where available, stored *M tuberculosis* isolates were recultured for DNA extraction. WGS was done on libraries prepared from purified genomic DNA using Illumina Nextera XT library (Illumina, San Diego, CA, USA) and NEBNext Ultra TM II FS DNA Library Prep Kits (New England Biolabs, Ipswich, MA, USA). Sequencing was done using the Illumina HiSeq 2500 or NextSeq 500 platforms. Sequencing data, with a minimum sequencing depth of 20× and 90% genomic coverage, were analysed using an in-house pipeline. Briefly, sequencing reads were mapped with Burrows-Wheeler Aligner to the inferred genome of the most recent common ancestor of the *M tuberculosis* complex (10·5281/zenodo.3497110) and fixed genomic variants were identified with VarScan2 (minimum frequency 90%). A pseudoalignment of concatenated polymorphic positions was generated excluding drug resistance positions and used to calculate the genetic distances between isolates using custom scripts. Isolates were clustered using the R package cluster with a single nucleotide polymorphism (SNP) distance threshold of five used to exclude direct transmission.^15^ To further assess the uniqueness of isolates, rifampicin-resistance conferring *rpoB* mutations were compared between unique isolates and the next closest isolate on the phylogeny. Raw FASTQ WGS data with a minimum coverage depth of 20× of the *M tuberculosis* H37Rv reference genome, were also analysed using TBProfiler (command line, version 2.8.12) to determine rifampicin-resistance conferring mutations and to identify strain lineages.^16^ The term isolate is used throughout to refer to patient specimens, although strain is used in reference to tuberculosis genotypic lineage.

### Data analysis

Both univariate and multivariate logistic regression analyses were done to assess associations between key factors and RMR tuberculosis in the whole cohort or genomically unique isolates in the WGS subcohort. Factors were entered into multivariate models based on univariate significance or presumed importance based on literature. χ^2^ analyses were used to assess trends over time, with the study period divided into two 5-year periods. All analyses were done with IBM SPSS Statistics, version 27. Classification of RMR tuberculosis versus MDR tuberculosis for the main cohort was based on routine diagnosis (primarily based on Xpert MTB/RIF or line probe assay results, or both). For the subcohort with available WGS data, classification was based on the WGS resistance profile (TBProfiler). Time from most recent previous tuberculosis treatment episode to rifampicin-resistant tuberculosis diagnosis was calculated based on treatment start date of the previous episode.

### Role of the funding source

The study funders had no role in the design and conduct of the study, the analysis and interpretation of data, or in the preparation, review, or approval of the manuscript.

## Results

**Figure 1:**
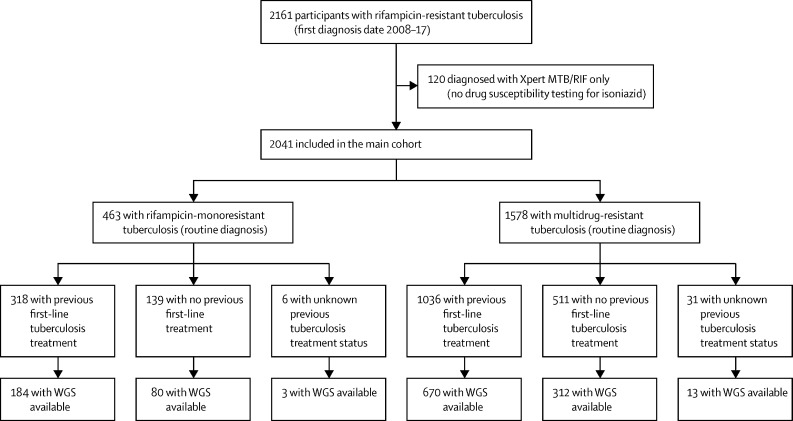
Study profile WGS=whole-genome sequencing.

**Figure 2:**
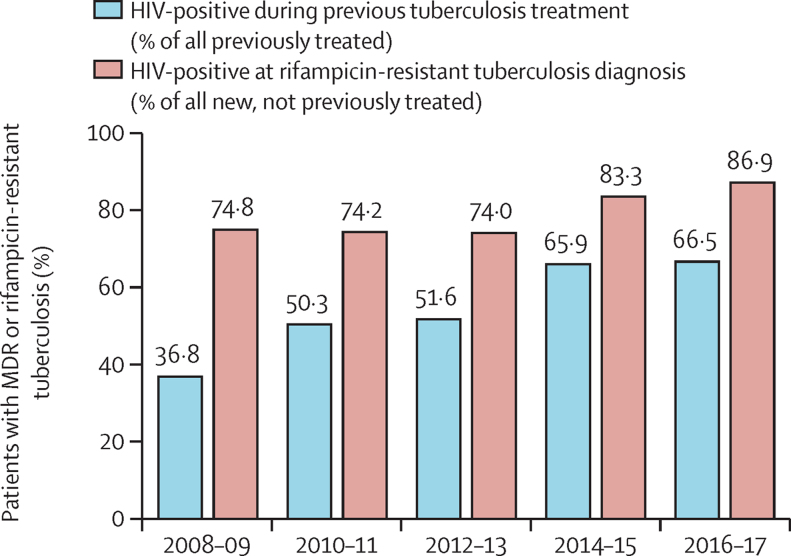
Percentage of previously treated patients with multidrug-resistant (MDR) or rifampicin-resistant tuberculosis who were HIV-positive during previous treatment and percentage of new patients with MDR or rifampicin-resistant tuberculosis who were HIV-positive at MDR or rifampicin-resistant tuberculosis diagnosis over the study time period

Overall, 2161 patients were diagnosed with microbiologically confirmed MDR or rifampicin-resistant tuberculosis between Jan 1, 2008, and Dec 31, 2017, in Khayelitsha, Cape Town; of those 120 (5·5%) were excluded as they were diagnosed solely with Xpert MTB/RIF without further testing for isoniazid resistance. This exclusion left 2041 patients in the main cohort, of whom 463 (22·7%) were diagnosed with RMR tuberculosis. Previous first-line tuberculosis treatment was identified in 1354 (66·3%) of 2041 patients (figure 1). In previously treated individuals, the percentage identified to have been HIV-positive during previous tuberculosis treatment increased across the study period (χ^2^ for trend, p<0·0001, figure 2). The percentage of new, not previously treated, individuals who were HIV-positive at MDR or rifampicin-resistant tuberculosis diagnosis also tended to increase over time, but this was not statistically significant (p=0·0001, figure 2).

On univariate analysis, diagnosis in the later time period of 2013–17, being HIV-positive at MDR or rifampicin-resistant tuberculosis diagnosis, and having three or more previous first-line tuberculosis treatment episodes were associated with diagnosis of RMR tuberculosis compared with MDR tuberculosis. In contrast, HIV-negative individuals with previous first-line tuberculosis treatment were less likely to have RMR tuberculosis than those with no previous tuberculosis treatment (table 1).

**Table 1:** Description of main cohort and univariate associations with RMR tuberculosis

	**Total (n=2041)**	**RMR tuberculosis (%)**	**Univariate OR (95% CI)**
**Year of MDR or rifampicin-resistant tuberculosis diagnosis**			
2008–12	1066	219 (20·5%)	1 (ref)
2013–17	975	244 (25·0%)	1·29 (1·05–1·59)
**Sex**			
Female	991	223 (22·5%)	1·02 (0·83–1·26)
Male	1050	240 (22·9%)	1 (ref)
**Age, years**			
≤24	319	76 (23·8%)	1 (ref)
25–34	744	184 (24·7%)	1·05 (0·77–1·43)
35–44	634	131 (20·7%)	0·83 (0·60–1·15)
≥45	344	72 (20·9%)	0·85 (0·59–1·22)
**HIV status at MDR or rifampicin-resistant tuberculosis diagnosis**			
HIV-negative	502	94 (18·7%)	1 (ref)
HIV-positive	1505	361 (24·0%)	1·37 (1·06–1·77)
Unknown	34	8 (23·5%)	1·34 (0·59–3·04)
**Previous tuberculosis treatment**			
No	650	139 (21·4%)	1 (ref)
Yes	1354	318 (23·5%)	1·13 (0·90–1·41)
Unknown	37	6 (16·2%)	0·71 (0·29–1·74)
**Number of previous tuberculosis treatment episodes**			
1	684	144 (21·1%)	1 (ref)
2	241	64 (26·6%)	1·36 (0·97–1·91)
≥3	92	34 (37·0%)	2·20 (1·38–3·49)
Unknown	337	76 (22·6%)	1·09 (0·80–1·50)
**Time between start of most recent previous tuberculosis treatment episode and MDR or rifampicin-resistant tuberculosis diagnosis**			
≤1 year	387	92 (23·8%)	1 (ref)
>1 year	630	151 (24·0%)	0·96 (0·81–1·14)
**HIV status during previous tuberculosis treatment**			
HIV-negative, no previous tuberculosis treatment	213	52 (24·4%)	1 (ref)
HIV-negative, previous tuberculosis treatment	287	43 (15·0%)	0·55 (0·35–0·86)
HIV-positive, no previous tuberculosis treatment	426	85 (20·0%)	0·77 (0·52–1·14)
HIV-positive during previous tuberculosis treatment	716	189 (26·4%)	1·11 (0·78–1·58)
HIV-positive at MDR or rifampicin-resistant tuberculosis diagnosis, unknown during previous tuberculosis treatment	291	69 (23·7%)	0·96 (0·64–1·46)
HIV-positive at MDR or rifampicin-resistant tuberculosis diagnosis, HIV-negative during previous tuberculosis treatment	50	12 (24·0%)	0·98 (0·48–2·01)
HIV status, or previous tuberculosis treatment status, or both, unknown	58	13 (22·4%)	0·89 (0·45–1·79)

Data are n, n (%), and OR (95% CI). RMR=rifampicin-monoresistant. OR=odds ratio. MDR=multidrug-resistant.

On multivariate analysis, in all patients with previous first-line tuberculosis treatment, three or more previous tuberculosis treatment episodes and being HIV-positive during previous tuberculosis treatment were independently associated with increased RMR tuberculosis relative to MDR tuberculosis. RMR tuberculosis was also associated with patients who were HIV-positive at MDR or rifampicin-resistant tuberculosis diagnosis but for whom HIV status during previous tuberculosis treatment was unknown, and those who were recorded as being HIV-negative during previous tuberculosis treatment. Finally, older age (35–44 years and ≥45 years) was associated with lower RMR tuberculosis compared with MDR tuberculosis (table 2).

**Table 2:** Multivariate logistic regression analysis of factors potentially associated with rifampicin-monoresistant tuberculosis in the main cohort of patients previously treated for tuberculosis

	**Multivariate OR (95% CI; n=1354)**
**Year of MDR or rifampicin-resistant tuberculosis diagnosis**	
2008–12	1 (ref)
2013–17	1·21 (0·92–1·58)
**Sex**	
Female	1·05 (0·81–1·37)
Male	1 (ref)
**Age, years**	
≤24	1 (ref)
25–34	0·75 (0·50–1·13)
35–44	0·55 (0·35–0·88)
≥45	0·58 (0·36–0·94)
**Number of previous tuberculosis treatment episodes**	
1	1 (ref)
2	1·27 (0·90–1·81)
≥3	1·96 (1·21–3·17)
Unknown	1·21 (0·75–1·95)
**HIV status during previous tuberculosis treatment**	
HIV-negative	1 (ref)
HIV-positive	2·07 (1·35–3·18)
HIV-positive at MDR or rifampicin-resistant tuberculosis diagnosis, unknown during previous tuberculosis treatment	1·84 (1·12–3·01)
HIV-positive at MDR or rifampicin-resistant tuberculosis diagnosis, HIV-negative during previous tuberculosis treatment	2·18 (1·01–4·68)
HIV status, or previous tuberculosis treatment status, or both, unknown	5·15 (1·38–19·25)

Data are OR (95% CI). OR=odds ratio. MDR=multidrug-resistant.

WGS data were available for 1262 (61·8%) of 2041 patients in the main cohort. WGS data were significantly more likely to be available from patients who were HIV-positive at MDR or rifampicin-resistant tuberculosis diagnosis, and from those routinely diagnosed with RMR tuberculosis, although these differences were small overall (appendix p 1). In 1262 patients with WGS data available, 93 isolates were classified as rifampicin-susceptible by TBProfiler and were excluded from further analysis, leaving 1169 isolates in the WGS subcohort. Of those 1169, 788 (67·4%) were identified as lineage 2, 346 (29·6%) as lineage 4, 28 (2·4%) as lineage 3, and seven (0·6%) as lineage 1. Phylogenetic trees for lineages 2 and 4 are given in the appendix (pp 2–3).

Using an SNP distance threshold of five, 360 (30·8%) of 1169 isolates were classified as genomically unique (not clustered with any other isolates). On univariate analysis, patients with unique isolates were more common: in the later time period, from those aged 35–44 years, from individuals who were HIV-positive at MDR or rifampicin-resistant tuberculosis diagnosis, those with three or more previous tuberculosis treatment episodes, those with RMR tuberculosis, and among isolates identified as lineages 1 and 4 (table 3).

**Table 3:** Description of WGS subcohort and univariate associations with unique isolates

	**Total (n=1169)**	**Unique (%)**	**OR (95% CI)**
**Year of MDR or rifampicin-resistant tuberculosis diagnosis**			
2008–12	642	178 (27·7%)	1 (ref)
2013–17	527	182 (34·5%)	1·38 (1·07–1·76)
**Sex**			
Female	565	183 (32·4%)	1·16 (0·90–1·48)
Male	604	177 (29·3%)	1 (ref)
**Age, years**			
≤24	198	48 (24·2%)	1 (ref)
25–34	425	128 (30·1	1·35 (0·92–1·98)
35–44	352	127 (36·1%)	1·76 (1·19–2·61)
≥45	194	57 (29·4%)	1·30 (0·83–2·04)
**HIV status at MDR or rifampicin-resistant tuberculosis diagnosis**			
HIV-negative	320	79 (24·7%)	1 (ref)
HIV-positive	834	276 (33·1%)	1·51 (1·13–2·02)
Unknown	15	5 (33·3%)	1·53 (0·51–4·50)
**Previous tuberculosis treatment**			
No	362	103 (28·5%)	1 (ref)
Yes	793	253 (31·9%)	1·18 (0·90–1·55)
Unknown	14	4 (28·6%)	1·01 (0·31–3·28)
**Number of previous tuberculosis treatment episodes**			
1	416	126 (30·3%)	1 (ref)
2	144	45 (31·3%)	1·05 (0·69–1·58)
≥3	39	20 (51·3%)	2·42 (1·25–4·70)
Unknown	194	62 (32·0%)	1·08 (0·75–1·56)
**Time between start of most recent previous tuberculosis treatment episode and MDR or rifampicin-resistant tuberculosis diagnosis**			
≤1 year	221	68 (30·8%)	1 (ref)
>1 year	378	123 (35·2%)	1·09 (0·76–1·55)
Unknown	194	62 (32·0%)	1·06 (0·70–1·60)
**HIV status during previous tuberculosis treatment**			
HIV-negative, no previous tuberculosis treatment	128	37 (28·9%)	1 (ref)
HIV-negative, previous tuberculosis treatment	190	42 (22·1%)	0·70 (0·42–1·17)
HIV-positive, no previous tuberculosis treatment	229	64 (27·9%)	0·95 (0·59–1·54)
HIV-positive during previous tuberculosis treatment	402	144 (35·8%)	1·37 (0·89–2·12)
HIV-positive at MDR or rifampicin-resistant tuberculosis diagnosis, unknown during previous tuberculosis treatment	164	56 (34·1%)	1·28 (0·77–2·10)
HIV-positive at MDR or rifampicin-resistant tuberculosis diagnosis, HIV-negative during previous tuberculosis treatment	33	10 (30·3%)	1·07 (0·46–2·47)
HIV status, or previous tuberculosis treatment status, or both, unknown	23	76 (30·4%)	1·08 (0·41–2·83)
**Drug resistance profile (WGS)**			
RMR tuberculosis	236	129 (54·7%)	3·66 (2·72–4·93)
MDR tuberculosis	933	231 (24·8%)	1 (ref)
**Strain lineage**			
Lineage 1	7	5 (71·4%)	7·87 (1·51–40·89)
Lineage 2	788	190 (24·1%)	1 (ref)
Lineage 3	28	8 (28·6%)	1·26 (0·55–2·91)
Lineage 4	346	157 (45·4%)	2·61 (2·00–3·42)

Data are n, n (%), and OR (95% CI). WGS=whole-genome sequencing. OR=odds ratio. MDR=multidrug-resistant. RMR=rifampicin-monoresistant.

Of RMR tuberculosis isolates, 55% (130 of 236) were unique compared with 25% (231 of 933) of MDR tuberculosis isolates (p<0·0001). In addition, there was a strong association between drug resistance profile and *M tuberculosis* strain lineage: 32% (112 of 346) of lineage 4 isolates were RMR tuberculosis compared with only 11·8% (93 of 788) of lineage 2 isolates. Given this association, only drug resistance profile was entered into multivariate models.

In previously treated patients, in a multivariate analysis, factors associated with unique isolates were: RMR tuberculosis versus MDR tuberculosis, age 35–44 years versus 24 years or younger, HIV positivity during previous first-line tuberculosis treatment versus HIV negativity during first-line treatment, and diagnosis in 2013–17 versus 2008–12 (table 4). Given the strong association with RMR tuberculosis (odds ratio 4·96, 95% CI 3·40–7·23), a separate multivariate analysis was done in previously treated patients with RMR tuberculosis; HIV positivity during previous tuberculosis treatment (5·13, 1·61–16·32) and female sex (2·50, 1·18–5·26) were associated with uniqueness, although three or more previous tuberculosis treatment episodes also tended to be associated with uniqueness (table 4). Of the 164 previously treated patients with RMR tuberculosis, 100 (61%) were classified as unique. Of these unique isolates, 91 (91%) were identified as having a different *rpoB* mutation to that in the closest isolate (identified based on phylogenetic analysis), supporting the inference of resistance acquisition.

**Table 4:** Multivariate analysis of factors potentially associated with unique *Mycobacterium tuberculosis* isolates in the subcohort of patients previously treated for tuberculosis and separately in the subcohort of patients with RMR tuberculosis

	**WGS subcohort (OR, 95% CI; n=793)**	**WGS subcohort with RMR tuberculosis (OR, 95% CI; n=164)**
**Year of MDR or rifampicin-resistant tuberculosis diagnosis**		
2008–12	1 (ref)	1 (ref)
2013–17	1·42 (1·02–1·99)	0·81 (0·39–1·69)
**Sex**		
Female	1·31 (0·94–1·82)	2·50 (1·18–5·26)
Male	1 (ref)	1 (ref)
**Age, years**		
≤24	1 (ref)	1 (ref)
25–34	1·09 (0·62–1·92)	2·37 (0·69–8·19)
35–44	1·78 (1·00–3·17)	2·66 (0·75–9·48)
≥45	1·43 (0·77–2·64)	0·94 (0·24–3·75)
**Drug resistance profile**		
MDR tuberculosis	1 (ref)	..
RMR tuberculosis	4·96 (3·40–7·23)	..
**Number of previous tuberculosis treatment episodes**		
1	1 (ref)	1 (ref)
2	0·84 (0·53–1·31)	0·65 (0·26–1·62)
≥3	1·48 (0·71–3·07)	4·32 (0·92–20·29)
Unknown	1·60 (0·92–2·78)	1·94 (0·54–7·04)
**HIV status during previous tuberculosis treatment**		
HIV-negative	1 (ref)	1 (ref)
HIV-positive	1·71 (1·03–2·84)	5·13 (1·61–16·32)
HIV-positive at MDR or rifampicin-resistant tuberculosis diagnosis, unknown during previous tuberculosis treatment	1·18 (0·47–2·98)	2·51 (0·63–9·91)
HIV-positive at MDR or rifampicin-resistant tuberculosis diagnosis, HIV-negative during previous tuberculosis treatment	1·18 (0·47–2·98)	1·69 (0·29–9·91)
HIV status unknown	0·93 (0·08–10·78)	..

RMR=rifampicin-monoresistant. WGS=whole-genome sequencing. OR=odds ratio. MDR=multidrug-resistant.

## Discussion

In this large cohort of patients diagnosed with MDR or rifampicin-resistant tuberculosis in Khayelitsha, Cape Town, over a 10-year period, HIV positivity during previous tuberculosis treatment was independently associated with both diagnosis of RMR tuberculosis compared with MDR tuberculosis and with identification of genomically unique *M tuberculosis* isolates based on WGS. In previously treated patients with RMR tuberculosis, HIV positivity during previous tuberculosis treatment was associated with a five-fold risk of genomic uniqueness. These data suggest that HIV infection during first-line tuberculosis treatment might be responsible for an increased risk of acquired rifampicin resistance, in turn leading to increased risk of subsequent RMR tuberculosis.

Since 2006, South Africa has seen a dramatic scale up of ART for HIV. Although this effort has contributed to decline in tuberculosis incidence overall, people living with HIV are likely to remain at higher risk of tuberculosis compared with HIV-negative individuals.^17^ This increased risk, combined with increased life expectancy, has resulted in increased numbers of people living with HIV developing tuberculosis and undergoing first-line tuberculosis treatment.^1^ The increasing proportion of patients who were HIV-positive during previous first-line tuberculosis treatment shown in Khayelitsha over time demonstrates this effect. Increasing numbers of people living with HIV with multiple episodes of tuberculosis treatment combined with even a small increased risk of acquired drug resistance could account for the increase in RMR tuberculosis observed in South Africa over time.^2^

Data supporting the role of HIV as a direct driver of the MDR or rifampicin-resistant tuberculosis epidemic has been inconsistent. Most studies show small positive associations in both new and previously treated cohorts of patients.^5^, ^6^ However, many of these studies were done in countries with a low tuberculosis burden, where there might be shared risk factors for MDR or rifampicin-resistant tuberculosis and HIV such as homelessness and substance misuse, compared with settings where HIV and tuberculosis are more prevalent. No association with HIV was seen in studies restricted to sub-Saharan African countries.^5^ Similarly, there was a trend towards a negative association between HIV and acquired resistance in two African studies included in a systematic review.^7^ These inconclusive data could be due to relatively small sample sizes in individual studies with insufficient power to detect small increases in the risk of resistance acquisition.

Historically, incomplete adherence to tuberculosis treatment has been linked to acquired drug resistance during tuberculosis treatment, with limited direct evidence.^18^ More recent data suggest that pharmacokinetic variability between individuals could be a larger contributor to resistance acquisition.^19^ A number of studies have described the emergence of resistance under strict adherence, particularly in people living with HIV.^20^ Poor adherence, in this setting, is therefore unlikely to explain the differences in association with RMR tuberculosis and risk of acquired resistance between HIV-positive and HIV-negative individuals. In contrast, data suggest that HIV, compounded by advanced immunosuppression and ART, might lead to lower concentrations of tuberculosis drugs and variable pharmacokinetics.^8^, ^21^ Individuals with low bodyweight, common in people living with HIV, might also have lower drug exposures due to higher proportional fat-free mass and weight-based dosing.^22^ However, a systematic review was unable to find a clear association between HIV and tuberculosis drug pharmacokinetics.^23^ As with describing associations between HIV and acquired resistance, this lack of association could also be due to insufficiently powered and heterogeneous studies. WHO target regimen profiles for tuberculosis suggest that new resistance to tuberculosis drugs should emerge in fewer than 1% of treatment episodes when taken as prescribed.^24^ However, few, if any, clinical trials are adequately powered to detect resistance acquisition at this level, and many trials to date have not enrolled individuals with advanced HIV. In addition, even the 1% level of resistance acquisition is predicted to result in continued high contributions of resistance acquisition to the MDR or rifampicin-resistant tuberculosis epidemic.^4^

While RMR tuberculosis is increasing in South Africa, lower genomic clustering in RMR tuberculosis strains, suggests that RMR tuberculosis might be less likely to be transmitted than MDR tuberculosis. The transmissibility of *M tuberculosis* strains is influenced by fitness costs associated with particular resistance conferring mutations.^25^ Previous data from this setting suggest that RMR tuberculosis isolates display a different profile of *rpoB* mutations conferring rifampicin resistance, including a significantly lower proportion of the common *rpoB* mutation Ser450Leu (unpublished data) that results in no or limited fitness cost to the bacteria, when compared with MDR tuberculosis.^25^ In addition, HIV infection has been shown to be associated with low-fitness *rpoB* mutations in a multicountry study of patients with both negative and positive HIV status.^26^ These data suggest that HIV could play an important role in the emergence of rifampicin-resistant and specifically RMR tuberculosis. It is also possible that inappropriate first-line treatment of RMR tuberculosis inhibits bacterial growth to a greater extent than it does for MDR tuberculosis, reducing individual infectiousness and therefore resulting in lower transmission. More detailed analysis of WGS data from this cohort will be required to discern variations in transmissibility by drug resistance profile, lineage, and patient-level factors.

In this study, we used WGS to determine genomically unique MDR or rifampicin-resistant tuberculosis isolates and genomic uniqueness to infer acquired drug resistance. Given that a considerable proportion of diagnosed MDR or rifampicin-resistant tuberculosis cases over the study period did not provide WGS data, and that an additional proportion of cases were likely to have been undiagnosed, non-clustering or uniqueness does not confirm that an individual was not directly infected with an MDR or rifampicin-resistant tuberculosis strain. However, such non-clustering does increase the likelihood of resistance acquisition as the cause of MDR or rifampicin-resistant tuberculosis and therefore can be used for analyses at a population level.^27^ This inference of acquired resistance was also supported by the observation that the majority of unique RMR tuberculosis isolates had different *rpoB* mutations when compared with the closest isolates in a phylogenetic tree. Of note, the diagnostic strategy for rifampicin-resistant tuberculosis changed over the study period, with universal drug susceptibility testing with Xpert MTB/RIF implemented from late 2011. Before this implementation, only individuals at high risk of rifampicin-resistant tuberculosis, predominantly those with previous tuberculosis treatment, received drug susceptibility testing. This change, which resulted in increased numbers of patients without previous tuberculosis treatment, could have introduced bias in the estimate of genomic uniqueness over time. However, this was a large sample of close to 1200 isolates from a geographically confined area over a decade. Although there are few large-scale studies describing associations between clustering at defined SNP thresholds and known epidemiological links, particularly in high tuberculosis burden settings and with respect to drug-resistant tuberculosis, use of a lower SNP distance threshold of five SNPs should efficiently exclude recent transmission.^15^

This study was also premised on the availability of high-quality retrospective data that could be used to assess whether patients with MDR or rifampicin-resistant tuberculosis were HIV-positive during previous first-line tuberculosis treatment. Although available data on HIV positivity from different data sources correlated well, there remains some uncertainty in the classification of patients who were known to be HIV-positive at MDR or rifampicin-resistant tuberculosis diagnosis, but were reported to be HIV-negative during previous tuberculosis treatment. These patients were primarily classified based on the recording of HIV negativity in a tuberculosis treatment register, without verification in any other data source. A proportion of these patients might have had undiagnosed HIV at the time of first-line tuberculosis treatment, potentially explaining the observed association with RMR tuberculosis in the multivariate analysis. A further limitation was the relatively poor quality and completeness of data on CD4 levels and ART during previous tuberculosis treatment. As a result, these factors, although relevant, were not included in these analyses.

Currently, tuberculosis treatment regimens for people living with HIV are largely the same as for those who are HIV-negative, and have changed little since the 1980s.^28^ However, evidence emerging over the last decade suggests that the rifampicin dose currently recommended for both HIV-negative and HIV-positive individuals could be insufficient and can be safely increased.^23^, ^29^ Encouragingly, there are several trials focused on increased rifampicin doses, including in people living with HIV.^30^ However, these trials, assessing pharmacokinetics, safety, and efficacy, are primarily focused on the potential for treatment shortening. That they will be sufficiently powered to quantify resistance acquisition overall or to detect any differences between HIV-negative and HIV-positive individuals is highly unlikely.

This study is novel as we have assessed the impact of HIV during previous tuberculosis treatment as opposed to HIV solely at the time of MDR or rifampicin-resistant tuberculosis diagnosis. Using these data, individuals who were HIV-positive during previous tuberculosis treatment were more likely to be infected with genomically unique *M tuberculosis* strains and these were more likely to be RMR tuberculosis compared with MDR tuberculosis. Although these data strongly suggest that HIV is driving the emergence of RMR tuberculosis during treatment in this particular setting, in the absence of clinical trials specifically addressing this issue, large-scale prospective cohort analyses across different patient populations are required to both directly quantify this additional risk and ultimately inform tuberculosis policies to mitigate the acquisition of rifampicin resistance.

## Data sharing

All sequencing data will be made available via online repository (European Nucleotide Archive) upon publication under accession number PRJEB45389. A limited deidentified dataset containing patient-level data will also be made available on publication.

## Declaration of interests

HC reports research grants from the US National Institutes of Health and the European and Developing Countries Clinical Trials Partnership outside the submitted work. All other authors declare no competing interests.
