# Supplementary material for: Potential contribution of HIV during first-line tuberculosis treatment to subsequent rifampicin-monoresistant tuberculosis and acquired tuberculosis drug resistance in South Africa: a retrospective molecular epidemiology study
Source: Lancet Microbe. 2021 Nov;2(11):e584–93. doi: 10.1016/S2666-5247(21)00144-0 (PMC8563432; doi:10.1016/S2666-5247(21)00144-0)

THE LANCET
Microbe

Supplementary appendix

This appendix formed part of the original submission and has been peer reviewed. We post it as supplied by the authors.

Supplement to: Cox H, Salaam-Dreyer Z, Goig GA, et al. Potential contribution of HIV during first-line tuberculosis treatment to subsequent rifampicin-monoresistant tuberculosis and acquired tuberculosis drug resistance in South Africa: a retrospective molecular epidemiology study. *Lancet Microbe* 2021; published online August 11. https://doi.org/10.1016/S2666-5247(21)00144-0.

HIV during first-line tuberculosis treatment is associated with rifampicin mono-resistant tuberculosis and acquired TB drug resistance: a retrospective molecular epidemiology study in South Africa

Supplementary material

Table 1: Availability of WGS data for main cohort patients.

	WGS data not available	WGS data available
N	779	1,262
MDR/RR-TB diagnosed 2013-17	389 (49.9%)	586 (46.4%)
Female	387 (49.7%)	604 (47.9%)
Median age (IQR)	34 (28-41)	33 (27-41)
HIV positive at MDR/RR-TB diagnosis	595 (76.4%)	910 (72.1%)
Previous TB treatment	500 (64.3%)	854 (67.7%)
RMR-TB (routine diagnosis)	196 (25.2%)	267 (21.2%)

Figure 1: Phylogenetic tree for lineage 2 isolates indicating genomically unique isolates at a single nucleotide polymorphism (SNP) threshold of 5 (first outer black ring) and 12 (second outer black ring). RMR-TB isolates are indicated in purple and MDR-TB isolates in red.

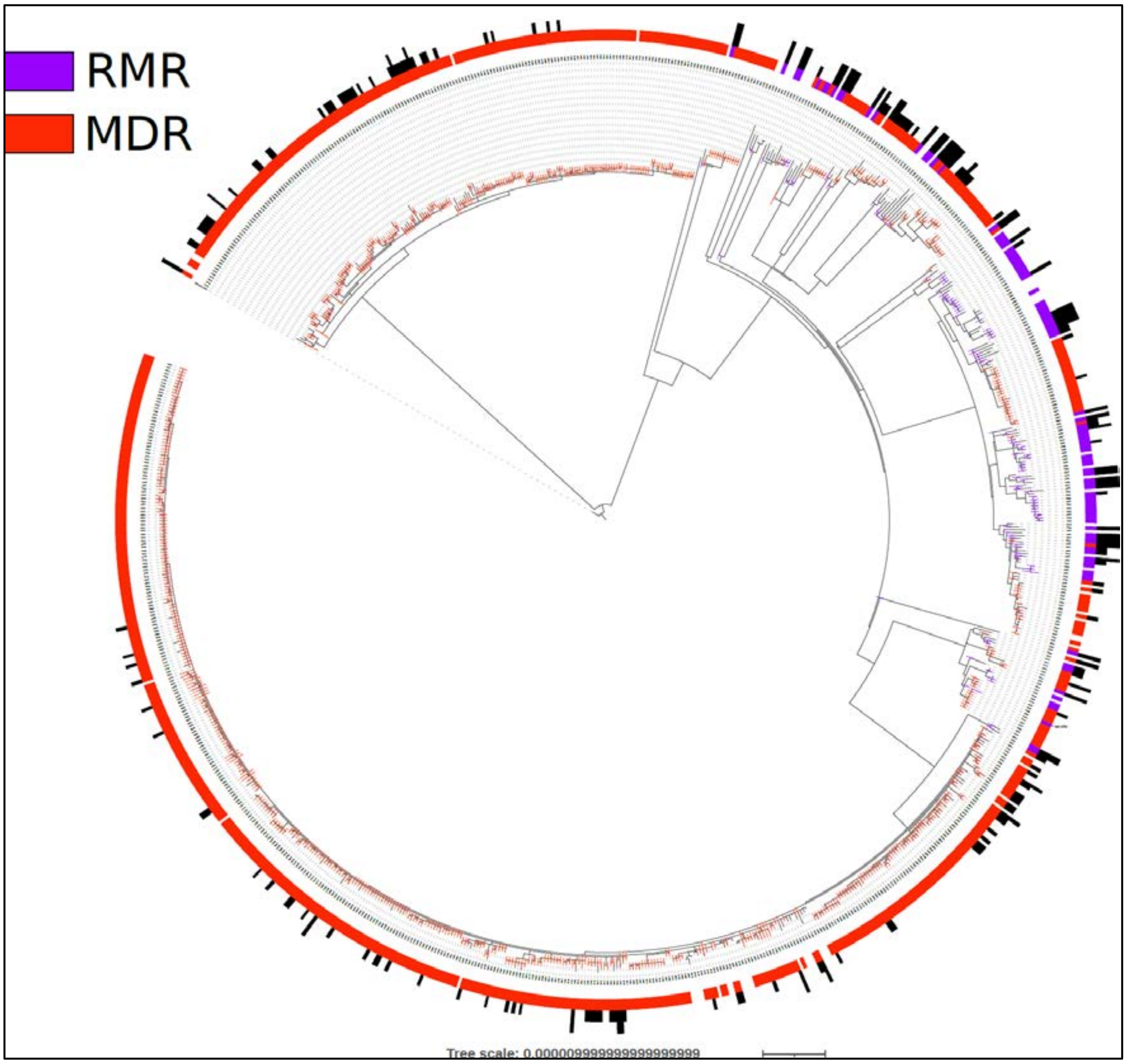

Figure 2: Phylogenetic tree for lineage 4 isolates indicating genomically unique isolates at a single nucleotide polymorphism (SNP) threshold of 5 (first outer black ring) and 12 (second outer black ring). RMR-TB isolates are indicated in purple and MDR-TB isolates in red.

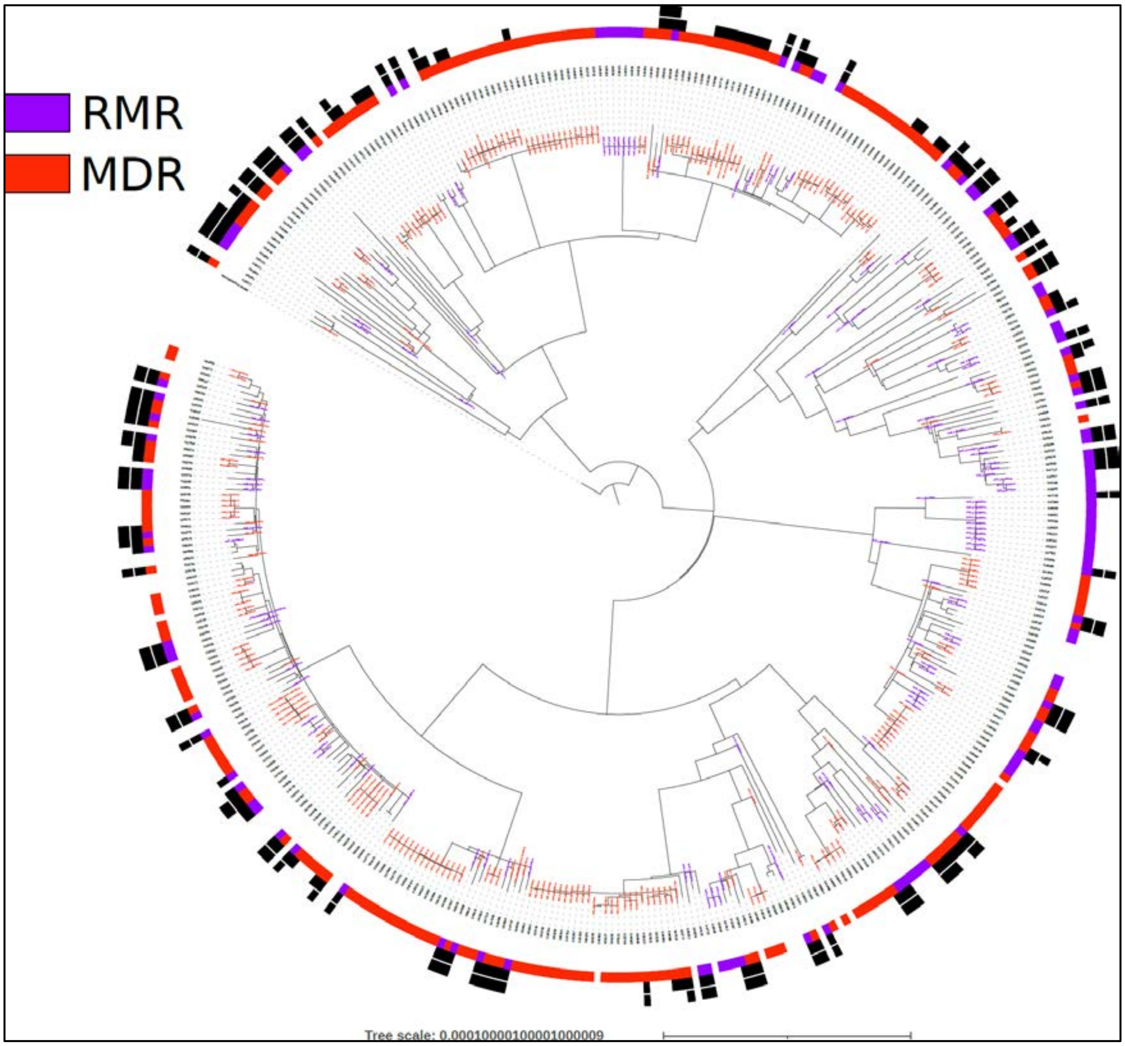

Supplement: Supplementary appendix [file mmc1.pdf]
